# Developmental dynamics of symptoms of emotional problems in childhood and adolescence: A longitudinal network analysis

**DOI:** 10.1002/jcv2.70079

**Published:** 2025-11-28

**Authors:** Eira R. Aksnes, Marianne Skogbrott Birkeland, Omid V. Ebrahimi, Mona Bekkhus, Lia Ferschmann, Dani Beck, Trude Fixdal, Alexandra Havdahl, Lydia Gabriela Speyer, Lars T. Westlye, Marieke G. N. Bos, Christian K. Tamnes

**Affiliations:** ^1^ Division of Mental Health and Substance Abuse Diakonhjemmet Hospital Oslo Norway; ^2^ Department of Psychology PROMENTA Research Center University of Oslo Oslo Norway; ^3^ Section for Implementation and Treatment Research Norwegian Centre for Violence and Traumatic Stress Studies Oslo Norway; ^4^ Department of Psychology University of Oslo Oslo Norway; ^5^ Department of Experimental Psychology University of Oxford Oxford UK; ^6^ Section for Violence and Abuse Norwegian Centre for Violence and Traumatic Stress Studies Oslo Norway; ^7^ Nic Waals Institute Lovisenberg Diaconal Hospital Oslo Norway; ^8^ PsychGen Centre for Genetic Epidemiology and Mental Health Norwegian Institute of Public Health Oslo Norway; ^9^ MRC Integrative Epidemiology Unit Population Health Sciences Bristol Medical School University of Bristol Bristol UK; ^10^ Institute for Early Life Care Paracelsus Medical University Salzburg Austria; ^11^ Center for Precision Psychiatry, Division of Mental Health and Addiction Oslo University Hospital Oslo Norway; ^12^ KG Jebsen Centre for Neurodevelopmental Disorders University of Oslo Oslo Norway; ^13^ Institute of Psychology Leiden University Leiden the Netherlands; ^14^ Leiden Institute for Brain and Cognition Leiden University Leiden the Netherlands

**Keywords:** ALSPAC, anxiety, depression, development, emotional problems, graphical vector autoregression

## Abstract

**Background:**

Epidemiological studies document increasing incident rates of mental disorders across childhood and adolescence, with mood and anxiety disorders particularly increasing among adolescent females. Research also indicates that these emotional problems have become more prevalent in recent decades. Yet, there is still a lack of understanding of the interrelated development of distinct emotional symptoms from childhood to adolescence.

**Methods:**

Here, we investigate and compare symptom dynamics in males and females. To accomplish this, we leveraged longitudinal data from the Avon Longitudinal Study of Parents and Children study (*N* = 11,120, 50.1% males at baseline). We used five items (*worries*, *unhappy*, *nervous*, *fearful*, *somatic complaints*) derived from the parent‐reported Strength and Difficulties Questionnaire emotional problems scale, measured at up to seven timepoints (mean age = 9.52, range = 4.0–18.3 years old). We estimated a panel Graphical Vector Autoregressive network model (GVAR) and statistically compared the networks of males and females.

**Results:**

The temporal network revealed largely reciprocal associations among symptoms, with the strongest edges between *fearful–nervous* and *unhappy–worries* (*β* = 0.04–0.06). The contemporaneous and between‐person networks showed similar structures, although the between‐person network exhibited fewer but stronger associations, reflecting more stable individual differences. Somatic complaints were weakly connected in the temporal network but more strongly associated in contemporaneous and between‐person networks. Network invariance testing indicated no significant sex differences in average network structure.

**Conclusion:**

The study delineates the developmental dynamics of emotional symptoms across childhood and adolescence, highlighting bidirectional influences between core symptoms of depression and anxiety, but did not find support for sex differences in their developmental interrelatedness.

## Key points

**What's known?**

- Emotional problems in childhood and adolescence are key predictors of later mental health issues. Prior research shows sex‐specific trajectories, with emotional disorders increasing in adolescence, especially among females.

**What's new?**

- This study is among the first to apply longitudinal symptom‐level network modelling from childhood to adolescence in a large, population‐based sample. It uniquely disentangles dynamic within‐person processes from stable between‐person patterns and formally tests sex differences in emotional symptom networks—going beyond sum scores and diagnostic categories.

**What's relevant?**

- Understanding how emotional symptoms evolve and interact across development and between sexes can inform more targeted prevention and intervention strategies.

## Graphical abstract

This study examined how emotional symptoms co‐develop from childhood to adolescence using longitudinal data from over 11,000 children and adolescents. Network analyses revealed reciprocal links between core symptoms of anxiety and depression, with no significant differences between males and females. Findings underscore the interconnected and stable nature of emotional difficulties across development.

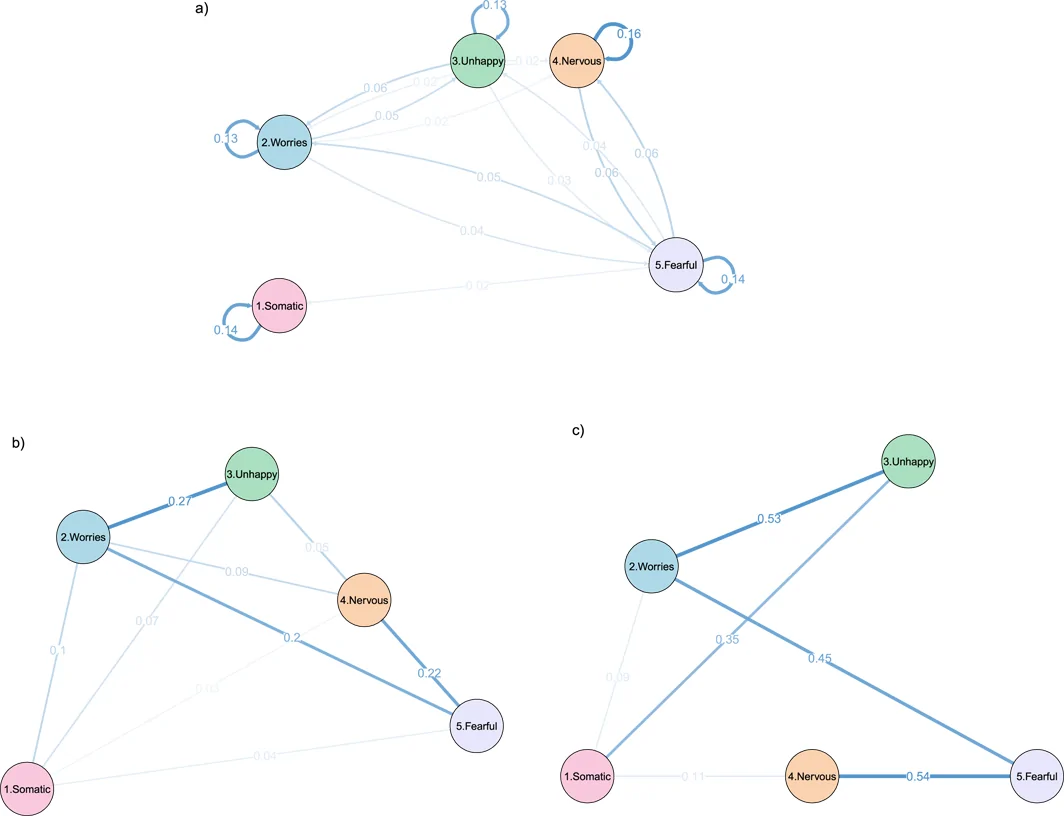

## INTRODUCTION

Mood and anxiety disorders increase substantially with age in late childhood and across adolescence, especially in females (Dalsgaard et al., 2020; Pasman et al., 2023). The prevalence of mental disorders doubles from 5–9 years old (6.81%) to 20–24 years old (13.63%), with sex‐specific patterns changing across development (Kieling et al., 2024). Female preponderance is seen in anxiety, depression and eating disorders with onset typically in adolescence, while males show higher rates of attention deficit hyperactivity disorder, conduct disorder and autism spectrum disorder with earlier onset (UNICEF, 2021). Emotional disorders such as depression and anxiety, particularly in girls, have increased notably in the past decades (Thapar et al., 2022). As mental health problems tend to accumulate across the lifespan (Caspi et al., 2014), and predict recurring disorders in adulthood (Johnson et al., 2018), early identification is crucial.

Developmental cascades theory (Masten & Cicchetti, 2010), posits that early symptoms ‘snowball’ over time into adolescence and adulthood (Moilanen et al., 2010). These cascading effects involve cumulative interactions within developing systems, including direct effects and indirect effects. In developmental psychopathology, cascades of adaptive and maladaptive symptoms are thought to shape development across levels and functions. Testing cascade models requires longitudinal data across domains, accounting for expected continuity covariance, and sufficient time for effects to unfold (Masten & Cicchetti, 2010). Most research has focused on broad dimensions or disorders rather than specific symptoms (Flouri et al., 2019), though findings suggest that early externalizing problems may cascade into later internalizing difficulties (Freichel et al., 2024).

In recent years there has been a shift in mental health research, from applying diagnostic categories to more dimensional perspectives (Conway et al., 2022). To better understand the emergence and development of children's emotional problems, it is important to consider the full range of symptom severity over time. Sub‐threshold symptoms are common in children and adolescents and may be precursors to later disorders (Bertha & Balázs, 2013). Prior work at the indicator level shows that *worry* and *unhappiness*, along with *stress handling* and *thinking clearly*, are particularly influential in adolescent mental health (Black et al., 2022). These findings underscore the value of symptom‐level investigations for adolescent mental health, as greater insight into how distinct emotional problem symptoms interact over time may aid in early detection and targeted intervention before problems become severe or chronic.

One approach increasingly used to address this complexity is the network theory of mental health (Borsboom, 2017; Borsboom et al., 2021; Ebrahimi, 2023). This framework views mental health problems as emergent from systems of interacting symptoms, rather than an underlying common cause. It accounts for comorbidity and heterogeneity by focusing on symptom‐level interactions (Cramer et al., 2010), which may be obscured by categorical diagnosis or sum scores (Ebrahimi et al., 2023; Fried, 2022). The statistical toolkit used to infer network structures or models from data, is referred to as network analysis (Bringmann et al., 2022).

Although, most network models have been estimated from cross‐sectional data in adult samples (Fried & Cramer, 2017), they can also be applied longitudinally using time‐series or panel data (Epskamp, 2020). Longitudinal models disaggregate between‐person and within‐person effects (Epskamp & Isvoranu, 2022), offering insight into how symptoms reinforce each other over time. These models go beyond snapshots of symptom co‐occurrence at a developmental stage, but also the dynamic process though which these patterns emerge.

Research applying this framework to child development remains limited, mainly focusing on the overall disorder level (Liu et al., 2023; Speyer, Hall, et al., 2022; Speyer, Ushakova, et al., 2022). Moreover, there is also a need to investigate whether males and females show different symptom dynamics across development, potentially clarifying disparities in prevalence, trajectories, and clinical features (Black et al., 2022).

In the present study, we examined longitudinal associations among specific emotional problem symptoms across childhood and adolescence, and whether these differed between males and females on average. We analysed parent‐reported item‐level data from the emotional problems scale of the Strength and Difficulties questionnaire (SDQ) across seven waves (approximately at 4, 7, 8, 9, 11, 13, 16 years of age) in the population‐based Avon Longitudinal Study of Parents and Children cohort (ALSPAC; Boyd et al., 2013; Fraser et al., 2013). Items included worries, nervousness, fearfulness, unhappiness, and somatic complaints, which capture core features of anxiety and depressive disorders and that have been validated as indicators of emotional disorders across development (Armitage, Tseliou, et al., 2023). We used a panel Graphical Vector Autoregression (panel‐GVAR) model to estimate temporal, contemporaneous, and between‐person networks. Sex‐differences were tested using a recently developed network invariance procedure (Hoekstra et al., 2024).

In line with the developmental cascade theory, we hypothesized that higher‐than average levels of emotional problems would predict higher levels at subsequent time points (i.e., a positive association). Consistent with findings from Black et al. (2022), we also hypothesized that *worry* and *unhappy* would represent the most central nodes for both between‐person and within‐person effects. Lastly, given the sex differences in prevalence of mood and anxiety disorders from childhood to adolescence, we explored whether symptom associations also would differ by sex. As prevalence differences do not necessarily imply differences in network couplings, these analyses were treated as exploratory.

## METHODS

### Sample

We used data from ALSPAC, a British longitudinal birth cohort study available for approved users. Pregnant women resident in Avon, UK with expected delivery dates from April 1991 to December 1992 were invited to participate in the study. The initial number of pregnancies enroled was 14,541 with 13,988 children alive at 1 year of age (Boyd et al., 2013; Fraser et al., 2013). The study website contains details of available data through a searchable data dictionary and variable search tool (http://www.bristol.ac.uk/alspac/researchers/our‐data/). The present study used parent‐reported SDQ data collected at ∼4, 7, 8, 9, 11, 13, and 16 years of age. Across these waves, 11,120 participants had at least one completed SDQ measure; 11,030 had at least one timepoint with full SDQ data (mean complete waves = 4.53, SD = 2.13, range 1–7; Figure S1). A total of 7519 (68.17%) had complete data at ≥4 timepoints. The final sample included 264,185 observations (50.13% male). ALSPAC's sex variable refers to sex assigned at birth.

### Ethical considerations

Ethical approval for the study was obtained from the ALSPAC Ethics and Law Committee and the Local Research Ethics Committees. Informed consent for the use of data collected via questionnaires and clinics was obtained from participants following the recommendations of the ALSPAC Ethics and Law Committee at the time. Further information is available elsewhere (https://www.bristol.ac.uk/alspac/researchers/research‐ethics/). The present study was approved by the ALSPAC Executive Committee, the Norwegian Regional Committees for Medical and Health Research Ethics, and the Norwegian Centre for Research Data.

### Emotional problem symptoms

We used the items from the emotional problems scale from the SDQ, a widely used, valid continuous measure of child mental health, suitable for distinguishing clinical and healthy groups (Goodman & Goodman, 2009). In addition, the SDQ has been validated as an appropriate measure to determine risk for emotional disorders like depression and anxiety among males and females across childhood and adolescence (Armitage et al., 2023). Items included were: ‘Many worries, often seems worried’ (*worries*), ‘Often unhappy, down‐hearted or tearful’ (*unhappy*), ‘Nervous or clingy in new situations, easily loses confidence’ (*nervous*), ‘Many fears, easily scared’ (*fearful*) and ‘Often complains of headache, stomach‐aches or sickness’ (*somatic complaints*). Each item is rated on a 3‐point Likert‐scale (*Not true*, *Somewhat true*, *Certainly true*). Higher scores indicate greater symptom burden. Items were rated based on the past 6 months or school year. Cronbach's alpha values for each wave are reported in Supplementary Table S12.

### Statistical analysis

We estimated panel‐GVAR models using the *psychonetrics* package and visualized results with the *qgraph* package (Epskamp et al., 2012) in R (version 4.3.1; R Core Team, 2024). Models were fitted for the full sample and by sex. The Graphical Vector Autoregressive (GVAR) model estimates temporal, contemporaneous and between‐person effects by separating within and between‐person effects by estimation of random intercepts, within‐person deviations from those intercepts, and the residual covariances as concurrent associations (Epskamp et al., 2018). As GVAR models assume stationarity, prior to model estimation, the data was detrended for linear and quadratic age trends and standardized across timepoints. This approach, which is common in developmental GVAR studies (Speyer et al., 2021), ensures that temporal dynamics reflect average within‐individual change rather than group‐level age‐related trends. To adhere to the model assumption of equidistant measures, we inserted a single dummy wave between wave 6 and 7 (ages 14 and 16), which had an unusually long interval (3.68 years), thereby aligning the spacing more closely with that of earlier waves (e.g., Freichel et al., 2025). Age summary by timepoint is provided in Table S1.

Model estimation leads to separation of variance into three separate networks. *The temporal network* highlights the predictive effect of one variable (also referred to as a node) on another variable at the next measurement occasion (i.e., over time), while controlling for all other nodes in the network. The temporal network includes directed edges (i.e., predictive effects over time), visualised as one headed‐arrowed lines, including both autocorrelations and cross‐lagged relations. This network is useful for understanding temporal dynamics between symptoms, for example, highlighting how one symptom increases another at the next time‐point, or whether a vicious cycle exists between two symptoms, amplifying each other over time (Ebrahimi et al., 2021; Speyer et al., 2022). *The contemporaneous network* reflects how momentary fluctuations in one variable is associated with momentary fluctuations in another variable at a given time, while controlling for both the temporal effects and all other nodes in the network (Bringmann et al., 2022; Epskamp, 2020). Finally, *the between‐person network* embodies effects on the between‐person level, highlighting how higher levels on a symptom compared to peers are associated with the mean levels of other symptoms compared to others in the population (Freichel & Epskamp, 2024).

To ensure that the model fitted the data appropriately we used different model fit statistics, specifically the Comparative Fit Index (CFI), Tucker‐Lewis index (TLI), and Root Mean Square Error of Approximation (RMSEA). Although these indices originate from SEM, their use in panel GVAR models is supported by recent work (Du et al., 2025), which recommends applying the same interpretive guidelines to evaluate model fit in this context.

See Supporting Information S1 for comment on applied fit indices (Appendix S1). The statistical models leveraged Full Information Maximum Likelihood (FIML) to address missing data, which enables inclusion of records with partially missing data. Use of FIML is considered state‐of‐the‐art in treating missing data, increasing statistical power and decreasing bias in comparison to complete‐case analysis (Enders & Bandalos, 2001). We also performed a missingness analysis to explore potential patterns of missingness in the data (Tables S2a  and S2b). Models were fitted across all seven timepoints. First, we estimated a saturated network model including all edges to examine whether a lag‐1 process may appropriately describe the data (Epskamp, 2020). The second step involved estimating a pruned network model where non‐significant edges are removed (using alpha = 0.05) to make the models robust against false positive conclusions. Model fit was evaluated after pruning, without reintroducing edges through a step‐up procedure (Blanken et al., 2022; Epskamp, 2020; Freichel, 2023).

To examine the relative importance of nodes in the temporal network, we computed the in‐strength (the extent to which node is predicted by other nodes at the previous timepoint) and out‐strength (the extent to which a node predicts other nodes at the next timepoint) of the different nodes (see Figure S2) We also computed strength centrality for the contemporaneous network (Figure S3). In addition, we performed a case‐dropping bootstrapping procedure to check for network stability (Epskamp et al., 2018).

To test for sex differences in the networks we used the network invariance test, which has been validated as a useful technique in detecting (in)difference between network structures (Hoekstra et al., 2024). Network invariance across sex was tested by comparing an equal and a freely estimated model using the Akaike Information Criterion (AIC), with lower AIC values indicating better fit.

## RESULTS

### Sample descriptives

Descriptive statistics (sample sizes, means, SDs), for all seven waves can be found in Tables S3–S9. To enhance the clinical characterization of the sample, we report the percentage of participant with a parent‐reported ICD‐10 diagnosis for any emotional, anxiety, or depressive disorder at child ages 7.5, 10 and 13 years in Table S10. Additionally, the SDQ categorizes emotional problem sum scores into a normal, borderline and abnormal range. Table S11 presents the distribution of participants within these categories, separately for males and females for each timepoint.

### Model fit

**FIGURE 1:**
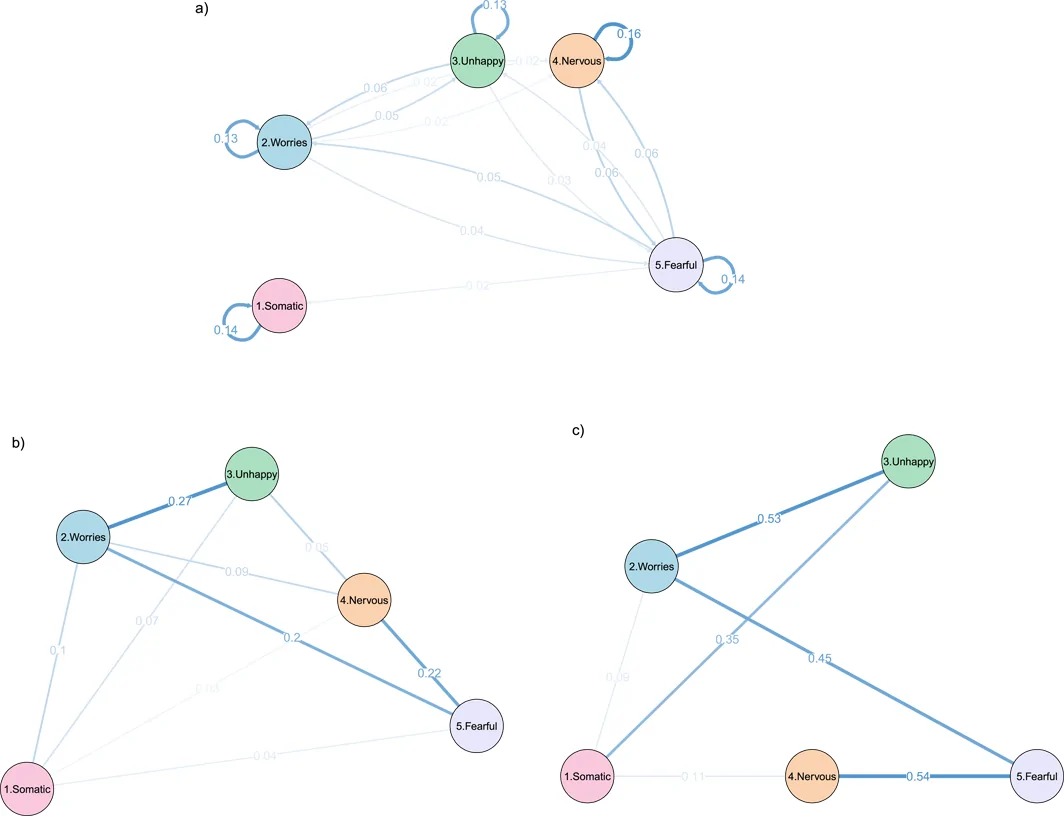
Full group networks. (A) The within‐person temporal Graphical Vector Autoregressive network standardized to directed partial correlations, (B) the within‐person contemporaneous partial correlation network, and (C) the between‐person partial correlation network. Blue edges (solid lines) indicate positive associations.

The saturated panel model provided an acceptable fit to the data (CFI = 0.88, TLI = 0.88, RMSEA = 0.036). The pruned model showed a similar fit to the data (CFI = 0.88, TLI = 0.89, RMSEA = 0.035). Figure 1 shows the sparse temporal, contemporaneous and between‐person networks. Bootstrapping revealed sufficient stability of most associations (Figures S4  and S5). One iteration was excluded from the correlation plot as the sampling process resulted in a matrix containing not available values, likely due to the amount of missing data introduced by the added dummy wave.

### Longitudinal within‐person associations

The temporal network revealed mostly bidirectional positive associations, with the strongest associations between *fearful‐nervous* and between *unhappy*‐*worries*, followed by *worries*‐*fearful*. *Unhappy* predicted *nervous* and *fearful* predicted *somatic complaints—*its only temporal link. All symptoms showed self‐loops indicating a cumulative within‐person effect (i.e., a child's worrying at one timepoint predicted an increase in worry at the next timepoint on average). Overall, the temporal network indicated reciprocal increases in symptoms (apart from somatic symptoms) over time, however with limited magnitude (most below 0.06). Node strength plots showed that *Fearful* had the highest out‐strength, with its increase amplifying *unhappy*, *worries*, and *nervous* to the largest extent. *Worries* and *fearful* had the highest in‐strength, primarily predicted by *unhappy* and *nervous*. Overall, the temporal network showed modest, reciprocal symptom amplification.

### Contemporaneous within‐person associations

The contemporaneous network showed positive associations between all symptoms, with the strongest associations between *unhappy‐worries, fearful‐nervous*, and *worries‐fearful*. This indicated that children that *worry more* also tend to feel more *unhappy* within the same time window. In contrast to the temporal network, the contemporaneous network showed associations between *somatic complaints* and all other symptoms in the network, indicating that somatic complaints were only associated with the remaining symptoms on a shorter timescale, but not to the same extent across development.

### Between‐person associations

The between‐person network showed the fewest, but strongest edges of all the three networks. It shared an overall similar architecture as the contemporaneous network. Here, *somatic complaints* were less connected to other nodes than in the contemporaneous network, but more so than the temporal network. Given its measurement of person‐specific means over time, the between‐person network highlights relationships between trait‐like stable tendencies between individuals across development.

### Sex specific networks of emotional problem symptoms

**FIGURE 2:**
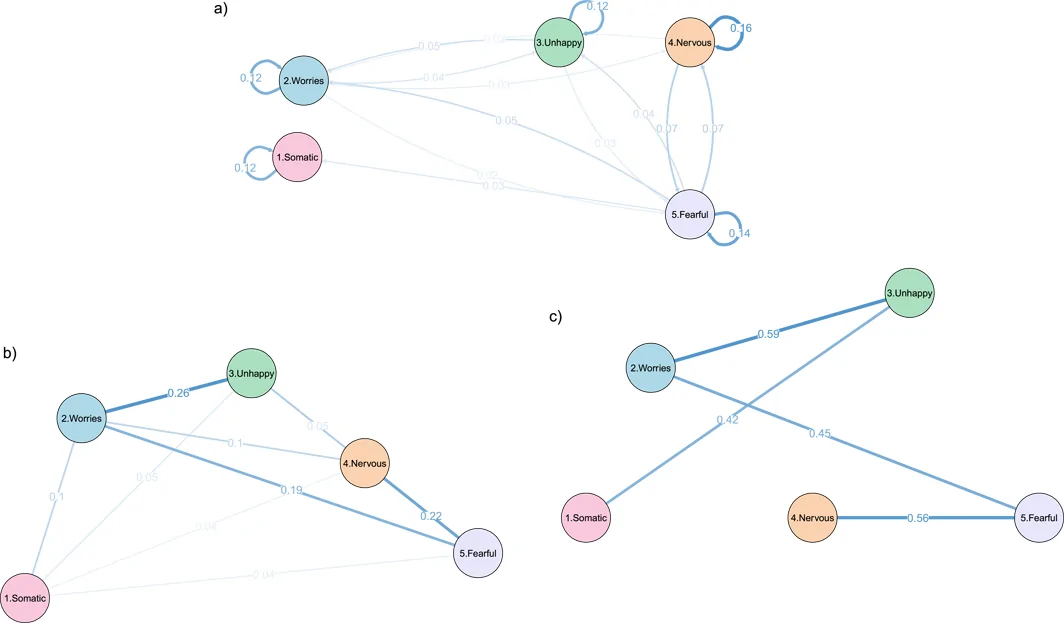
Male networks. (A) Fixed‐effect within‐person temporal Graphical Vector Autoregressive network standardized to directed partial correlations, (B) fixed‐effect within‐person contemporaneous partial correlation network, and (C) random‐effects between‐person partial correlation network. Blue edges (solid lines) indicate positive associations.

The saturated networks for males showed acceptable fit (CFI = 0.86, TLI = 0.86, RMSEA = 0.038), as did the pruned model (CFI = 0.86, TLI = 0.86, RMSEA = 0.038). The male temporal network largely mirrored the full sample, with bidirectional associations between most symptoms, except for a unidirectional effect from *fearful* to *somatic complaints* and no link between *nervous* and *unhappy*. All edges were positive. The contemporaneous network showed associations among all symptoms, with a structure similar to the full sample. The male between‐person network displayed the strongest associations, linking *worries‐unhappy, nervous‐fearful*, *fearful‐worries,* and *unhappy‐somatic complaints* (Figure 2).

**FIGURE 3:**
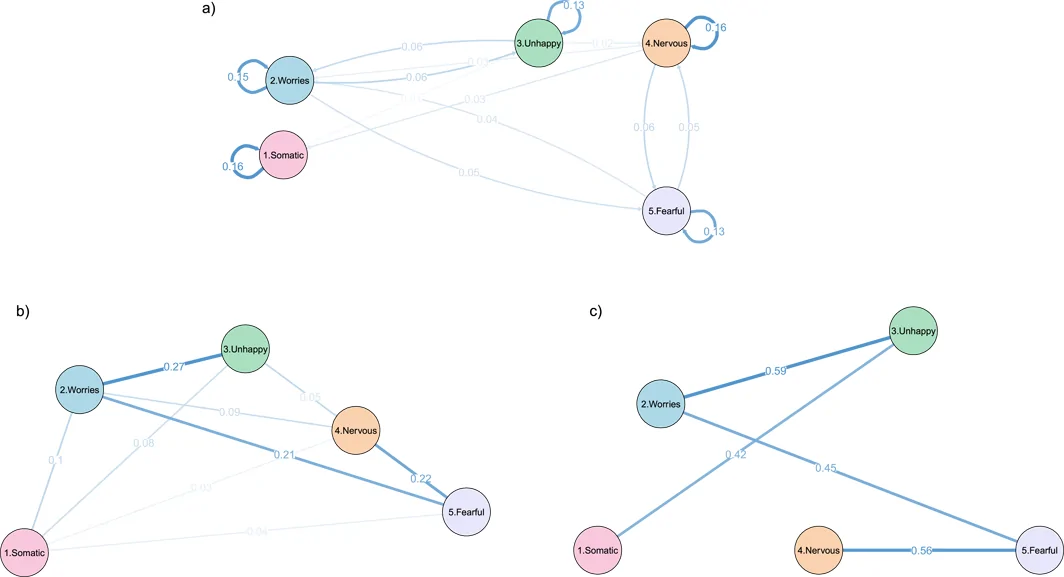
Female networks. (A) Fixed‐effect within‐person temporal Graphical Vector Autoregressive network standardized to directed partial correlations, (B) fixed‐effect within‐person contemporaneous partial correlation network, and (C) random‐effects between‐person partial correlation network. Blue edges (solid lines) indicate positive associations.

For females, both the saturated (CFI = 0.88, TLI = 0.88, RMSEA = 0.037), and pruned model (CFI = 0.88, TLI = 0.88, RMSEA = 0.036) showed an acceptable fit. The temporal female network showed bidirectional links between *nervous‐fearful, worries‐unhappy*, and *fearful‐worries.* Along with directed edges from *nervous* to *somati*c *complaints* and from *somatic complaints* to *unhappy.* The female contemporaneous network resembled the temporal network, but included stronger associations and more connections to *somatic complaints*. The female between‐person network had a similar structure to the within‐person networks, but with fewer and stronger associations (Figure 3). The network invariance yielded an AIC of 680,340.24 for the Difference model and an AIC of 680,320.01 for the Equal model, indicating no significant differences between male and female networks. This result was replicated for the pruned networks.

## DISCUSSION

This study employed a longitudinal network analysis approach to investigate within‐person and between person associations among emotional problem symptoms across childhood and adolescence. We hypothesized that higher than average levels of emotional problems would predict higher levels at subsequent time points (i.e., a positive associations) and that the structure of longitudinal symptom networks would differ between males and females. Overall, findings supported the first hypothesis: symptoms were positively associated both within and across timepoints and highlight the deleterious interconnectedness between symptoms aggravating each other over time. However, contrary to expectations, we found no significant differences in the average networks of males and females.

Across temporal, contemporaneous, and between‐person networks, we found stronger associations between symptoms typically grouped in within mood (*unhappy‐worries*) and anxiety disorders (*fearful‐nervous*), than across these domains. Unlike prior work, which has typically examined emotional problems at the subscale or diagnostic level, our approach allowed us to disentangle dynamic, short‐term within‐person fluctuations from longer‐term, trait‐like covariation at the symptom level. This granularity provides a more nuanced picture of symptom co‐development across childhood and adolescence, highlighting processes that may otherwise be obscured by broader diagnostic categories.

Overall, these findings align with developmental cascade theory and corroborate previous findings at both the indicator and subscale level (Black et al., 2022; Freichel et al., 2025). This suggests that emotional problem symptoms reinforce each other over time, creating self‐perpetuating vicious cycles. While edge weights were modes, this is expected in autoregressive models that account for stability effects and the inherent persistence of symptoms over time (Adachi & Willoughby, 2015). While model fit values (CFI and TLI) were slightly below conventional SEM thresholds, recent simulation work (Du et al., 2025) suggests these criteria may be overly conservative for complex panel network models, though some degree of interpretative caution is warranted.

### Temporal effects

The temporal network, capturing average within‐person effects over time, revealed moderate autoregressive associations (self‐loops) for all symptoms, particularly for *nervous*, *fearful* and *somatic complaints* (edge weights: 0.14–0.16), which is in line with former work at both the symptom level (Black et al., 2022) and the subscale level (Speyer et al., 2022). This finding indicates that the symptoms are fairly stable over time, with a tendency to persist within the same individual. *Fearful* emerged as the most influential symptom, predicting increases in other symptoms, and was the only node showing a positive, albeit weak, link to *somatic complaints* (0.02). This contrasts previous findings of Black et al. (2022) and our second hypothesis, who identified *unhappy* and *worry* to be the most central nodes in their network. However, their network involved a larger number of nodes as well as different assessment scales, making direct comparison difficult (Bringmann et al., 2022). Although the reciprocal associations between items were generally modest, they indicate meaningful co‐development among symptoms over time. These findings are consistent with developmental cascade models (Masten & Cicchetti, 2010), which posit that difficulties in one domain may spill over and reinforce difficulties in others. The persistent ties between fearfulness, worry, and unhappiness may reflect an underlying negative affectivity construct, supporting theories suggesting early emotion dysregulation as a transdiagnostic vulnerability (Eisenberg et al., 2001). It is important to note that these temporal associations are based on parent reports, which may therefore be more sensitive to observable behaviours such as nervousness or fearfulness than internal states like unhappiness.

### Contemporaneous effects

The contemporaneous network, which captures partial correlations between symptoms within the same timepoint, while controlling for temporal effects, revealed strong co‐activation among core symptoms at the same moment in time. The strongest associations emerged between *worries* and *unhappy* (0.27), *fearful* and *nervous* (0.22), and *fearful* and *worries* (0.20), suggesting tight clustering of anxiety related symptoms within a given developmental window. These patterns highlight *fearfulness* as a central node in short‐term symptom co‐occurrence. Many edges overlapped with those in the temporal network, but were stronger in magnitude, reflecting more immediate symptom interaction. Interestingly, *somatic complaints* were more connected to other symptoms (e.g., 0.10 with *worries*, 0.07 with *unhappy*) compared to the temporal network. This suggests that somatic issues may co‐occur with emotional problems on a shorter timescale (here within approximately 1–2 years' timescale), but are less integrated into the broader temporal emotional symptom network.

### Between‐person effects

The between‐person network, capturing partial correlations among individuals' average symptom levels across time, reveals which symptoms tend to co‐occur consistently across people. Between‐person associations likely reflect more stable vulnerabilities, such as temperament (i.e., neurotic tendencies), persistent environmental exposures (i.e., childhood maltreatment), or early neurobiological risk factors (i.e., genetic risk). These stable factors may predispose individuals to co‐occurring emotional symptoms, regardless of short‐term fluctuations. Importantly, such trait‐like associations could help identify youth at heightened long‐term risk, informing early, preventative interventions (Fried & Robinaugh, 2020).

The between‐person network was the sparsest (i.e., fewest edges) but exhibited the strongest associations. The most robust link was between *fearful* and *nervous* (0.54), suggesting that individuals who are generally nervous also tend to be generally more fearful. Strong trait‐level associations were also found between *worries and unhappy* (0.53), suggestive of stable anxiety tendencies (Boschloo et al., 2015). Notably, *somatic complaints* showed their strongest associations in this network, particularly with *unhappy* (0.35), which may indicate a somatization pattern not captured by shorter‐term associations. Together, these findings suggest that emotional problem symptoms, especially *fearfulness*, *worry* and *unhappiness,* cluster in trait‐like patterns across individuals, pointing to a subgroup of children and adolescents with heightened global emotional vulnerability. Again, as these associations are derived from parental ratings, they may reflect broader, trait‐like impressions of a child's emotional functioning, which might differ from self‐perceptions, especially in adolescence.

### Non‐specific sex‐effects

Given the known sex‐disparities in the prevalence and development of mood and anxiety disorders during childhood and adolescence, we explored whether symptom associations would differ by sex. Our findings suggest no differences in the developmental networks of emotional problems in males and females on average, contrary to our hypothesis.

Several factors may help explain this finding. Firstly, sex‐specific symptom dynamics might unfold over shorter timescales or in more nuanced ways than can be captured by our current model (Haslbeck & Ryan, 2022). Notably, our analysis compares *average* networks of males and females, without accounting for heterogeneity within each group. Recent work (e.g., Bolger et al., 2019; Chaku & Beltz, 2022) emphasizes that psychological processes may differ considerably among individuals within the same demographic category. Accordingly, future studies employing idiographic methods or intensive longitudinal designs may be better suited to detect such individual‐level dynamics (Russell & Gajos, 2020).

In line with this, Black et al. (2022) suggested that the absence of sex differences in developmental networks may reflect qualitative rather than quantitative differences in symptom expression. Quantitative differences refer to variations in the strength of associations or symptom severity, whereas qualitative differences involve different patterns or types of symptom interrelations. For example, girls and boys may both experience anxiety and sadness, but these may manifest in different behavioural expressions or be influenced by different contextual factors (e.g., peer dynamics, parental factors; Do et al., 2023; Höltge et al., 2022). Additionally, sex‐specific processes such as hormonal changes, socialization patterns, or coping strategies may emerge over shorter windows (e.g., during puberty), which may not be captured by biennial measurements (Crone & Dahl, 2012; Olivier et al., 2022).

In contrast to our findings, recent research using large and more recent longitudinal datasets have reported notable sex differences in the developmental networks of externalizing and internalizing difficulties (Liu et al., 2023). However, such differences may not manifest clearly at the symptom level and might only emerge when examining broader indicator‐level patterns (Speyer et al., 2022). It is also plausible that males and females share the same underlying symptom ‘wiring’, while differing primarily in prevalence, incidence, or overall risk load rather than in the structure of symptom interactions. Finally, it is worth considering that the ALSPAC cohort, launched in the early 1990s, may not fully reflect current sex‐disparities in mental health and more recent cohorts may yield different insights (Armitage, Kwong, et al., 2023).

### Practical implications and future directions

From a clinical standpoint, our findings underscore the value of symptom‐level assessments, which might inform early identification and intervention. The centrality of fearfulness and its predictive role in symptom escalation suggests that targeting fear responses in childhood in interventions (i.e., cognitive therapies, exposure‐based therapies or family‐based therapies) could mitigate broader or spiralling emotional difficulties later on. Integrating dynamical network models into applied settings has also been suggested as a way of tailoring care, by highlighting specific symptom constellations that warrant attention (Burger et al., 2022; Ebrahimi et al., 2023). More broadly, this symptom‐level approach highlights emotional problems as reinforcing, co‐evolving systems, and offers a nuanced understanding of how symptoms persist and escalate across development. These insights may ultimately support more precise prevention strategies and more effective timing of interventions.

### Limitations

This study offers novel insights into the dynamics of emotional problems from childhood to adolescence using a large, population‐based sample. However, several limitations should be considered. Firstly, our analyses are based on sample‐average networks, which may mask individual differences in emotional development. Future research should consider person‐specific or subgroup approaches to better capture this heterogeneity (Cusack et al., 2024; De Smet et al., 2024).

Secondly, the temporal resolution of the SDQ (i.e., recall over the past 6 months) limits the ability to capture more rapid fluctuations in emotional problems that may occur at daily or weekly scales. Future studies using more intensive, high‐frequency assessments could offer more granular insight into short‐term symptom dynamics. Nonetheless, our findings are informative about the slower paced changes in emotional problems unfolding across months or years, illuminating one aspect of developmental trajectories and emotional problems (McLaughlin et al., 2015). Retrospective bias is also a consideration when interpreting these findings, as it may affect the accuracy of symptom reports. Moreover, the dynamics of emotional problems may differ depending on the temporal granularity of measurement, where shorter intervals between assessment waves could reveal within‐person fluctuations, while longer intervals may better capture between‐person variability (Bringmann et al., 2022). Future studies could vary the spacing between assessments to capture different symptom dynamics, but this would require measures with shorter recall periods than the SDQ's 6‐month frame.

Third, all emotional problem symptoms were reported by parents across all waves, which may become less appropriate as children age, given the increasing salience of internal experiences that are less observable. While self‐report may improve accuracy in adolescence, shifting reporters across time brings comparability challenges.

Fourth, as with any prospective cohort study, ALSPAC experienced attrition over time, particularly among families facing greater adversity during pregnancy, potentially leading to underestimation of outcomes of interest. Although imputation techniques address some of this bias (Fraser et al., 2013), missingness remains a concern. In our sample, 30% of the participants had between one and three measurement points, which we acknowledge entails not an unsubstantial proportion of missingness, which in turn might influence the robustness of the results.

Fifth, we used a 3‐point Likert scale in the panelGVAR estimation, which treats responses as continuous. While this is not ideal, research suggests that in large samples, treating ordinal variables as continuous yields comparable results (Johal & Rhemtulla, 2023; Mircioiu & Atkinson, 2017; Robitzsch, 2020).

Sixth, our modelling approach does not detect non‐linear effects, which may be important in understanding complex developmental trajectories (Freichel, 2023). Finally, the SDQ items were zero‐inflated or exhibited non‐normal distributions, which is common in population‐based samples (Figures S6–S10). Although the GVAR model is relatively robust to such deviations, violations of the normality assumption may influence the precision and interpretation of the parameter estimates (Epskamp, 2020).

In general, only considering symptom level dynamics also omits important connections between symptoms and potential antecedents (socioeconomic risk factors, family factors, early life stress etc.), that are relevant in emotional problem development (Armitage et al., 2024). The integration of different levels of variables in multilayer networks has been proposed moving forward (Ebrahimi, 2023). Accordingly, a clear understanding of developmental systems may require an aggregation of evidence across multiple studies and methodological approaches.

### Conclusion

This study explored how specific emotional symptoms interrelate across childhood and adolescence and examined potential sex differences. Fearfulness consistently emerged as a central symptom, linking multiple emotional difficulties. Worries showed strong contemporaneous, but weaker temporal associations, suggesting it signals situational co‐occurrence rather than long‐term progression. Stronger between‐person than within‐person associations underscore the role of stable, trait‐like vulnerabilities in shaping emotional development. Contrary to expectations, no average sex differences were found in symptom dynamics. Future research integrating biological and social factors across varying timescales may improve understanding of individual mental health trajectories and support more targeted prevention strategies.

## AUTHOR CONTRIBUTIONS

**Eira R. Aksnes**: Conceptualization; formal analysis; investigation; methodology; project administration; validation; visualization; writing—original draft; writing—review and editing. **Marianne Skogbrott Birkeland**: Conceptualization; formal analysis; methodology; resources; supervision; writing—review and editing. **Omid V. Ebrahimi**: Conceptualization; formal analysis; methodology; resources; software; supervision; validation; writing—review and editing. **Mona Bekkhus**: Conceptualization; writing—review and editing. **Lia Ferschmann**: Resources; supervision; writing—review and editing. **Dani Beck**: Conceptualization; formal analysis; investigation; methodology; project administration; resources; software; supervision; visualization; writing—original draft; writing—review and editing. **Trude Fixdal**: Conceptualization; writing—review and editing. **Alexandra Havdahl**: Data curation; funding acquisition; methodology; resources; writing—review and editing. **Lydia Gabriela Speyer**: Conceptualization; formal analysis; methodology; resources; software; writing—review and editing. **Lars T. Westlye**: Conceptualization; funding acquisition; writing—review and editing. **Marieke G. N. Bos**: Conceptualization; resources; writing—review and editing. **Christian K. Tamnes**: Conceptualization; data curation; formal analysis; funding acquisition; investigation; methodology; project administration; resources; software; supervision; validation; visualization; writing—original draft; writing—review and editing.

## CONFLICT OF INTEREST STATEMENT

The authors declare no conflicts of interest.

## ETHICAL CONSIDERATIONS

Ethical approval for the study was obtained from the ALSPAC Ethics and Law Committee and the Local Research Ethics Committees. Informed consent for the use of data collected via questionnaires and clinics was obtained from participants following the recommendations of the ALSPAC Ethics and Law Committee at the time. Further information is available elsewhere (https://www.bristol.ac.uk/alspac/researchers/research‐ethics/). The present study was approved by the ALSPAC Executive Committee (24 June 2024, #B4645), the Norwegian Regional Committees for Medical and Health Research Ethics (25 June 2021, #269241), and the Norwegian Centre for Research Data (21 July 2021, #440199).

## Supporting information

Supporting Information S1

## Data Availability

The informed consent obtained from ALSPAC participants does not allow the data to be made available through any third party maintained public repository. Supporting data are available from ALSPAC on request under the approved proposal number, B4645. Full instructions for applying for data access can be found here: http://www.bristol.ac.uk/alspac/researchers/access/. The ALSPAC study website contains details of all available data (http://www.bristol.ac.uk/alspac/researchers/our‐data/).

## References

[jcv270079-bib-0001] Adachi, P. , & Willoughby, T. (2015). Interpreting effect sizes when controlling for stability effects in longitudinal autoregressive models: Implications for psychological science. European Journal of Developmental Psychology, 12(1), 116–128. 10.1080/17405629.2014.963549

[jcv270079-bib-0002] Armitage, J. M. , Collishaw, S. , & Sellers, R. (2024). Explaining long‐term trends in adolescent emotional problems: What we know from population‐based studies. Discover Social Science and Health, 4(1), 14. 10.1007/s44155-024-00076-2

[jcv270079-bib-0003] Armitage, J. M. , Kwong, A. S. F. , Tseliou, F. , Sellers, R. , Blakey, R. , Anthony, R. , Rice, F. , Thapar, A. , & Collishaw, S. (2023). Cross‐cohort change in parent‐reported emotional problem trajectories across childhood and adolescence in the UK. The Lancet Psychiatry, 10(7), S221503662300175X. 10.1016/S2215-0366(23)00175-X 37244272

[jcv270079-bib-0004] Armitage, J. M. , Tseliou, F. , Riglin, L. , Dennison, C. , Eyre, O. , Bevan Jones, R. , Rice, F. , Thapar, A. K. , Thapar, A. , & Collishaw, S. (2023). Validation of the Strengths and Difficulties Questionnaire (SDQ) emotional subscale in assessing depression and anxiety across development. PLoS One, 18(7), e0288882. 10.1371/journal.pone.0288882 37467238 PMC10355443

[jcv270079-bib-0005] Bertha, E. A. , & Balázs, J. (2013). Subthreshold depression in adolescence: A systematic review. European Child & Adolescent Psychiatry, 22(10), 589–603. 10.1007/s00787-013-0411-0 23579389

[jcv270079-bib-0006] Black, L. , Panayiotou, M. , & Humphrey, N. (2022). Internalizing symptoms, well‐being, and correlates in adolescence: A multiverse exploration via cross‐lagged panel network models. Development and Psychopathology, 34(4), 1477–1491. 10.1017/S0954579421000225 34128457

[jcv270079-bib-0007] Blanken, T. F. , Isvoranu, A.‐M. , & Epskamp, S. (2022). Estimating network structures using model selection. In Network psychometrics with R: A guide for behavioral and social scientists (pp. 111–132). Routledge. 10.4324/9781003111238

[jcv270079-bib-0008] Bolger, N. , Zee, K. S. , Rossignac‐Milon, M. , & Hassin, R. R. (2019). Causal processes in psychology are heterogeneous. Journal of Experimental Psychology: General, 148(4), 601–618. 10.1037/xge0000558 30973259

[jcv270079-bib-0009] Borsboom, D. (2017). A network theory of mental disorders. World Psychiatry, 16(1), 5–13. 10.1002/wps.20375 28127906 PMC5269502

[jcv270079-bib-0010] Borsboom, D. , Deserno, M. K. , Rhemtulla, M. , Epskamp, S. , Fried, E. I. , McNally, R. J. , Robinaugh, D. J. , Perugini, M. , Dalege, J. , Costantini, G. , Isvoranu, A.‐M. , Wysocki, A. C. , van Borkulo, C. D. , van Bork, R. , & Waldorp, L. J. (2021). Network analysis of multivariate data in psychological science. Nature Reviews Methods Primers, 1(1), 58. 10.1038/s43586-021-00055-w

[jcv270079-bib-0011] Boschloo, L. , Van Borkulo, C. D. , Rhemtulla, M. , Keyes, K. M. , Borsboom, D. , & Schoevers, R. A. (2015). The network structure of symptoms of the diagnostic and statistical manual of mental disorders. PLoS One, 10(9), e0137621. 10.1371/journal.pone.0137621 26368008 PMC4569413

[jcv270079-bib-0012] Boyd, A. , Golding, J. , Macleod, J. , Lawlor, D. A. , Fraser, A. , Henderson, J. , Molloy, L. , Ness, A. , Ring, S. , & Davey Smith, G. (2013). Cohort profile: The ‘Children of the 90s’—The index offspring of the Avon Longitudinal Study of Parents and Children. International Journal of Epidemiology, 42(1), 111–127. 10.1093/ije/dys064 22507743 PMC3600618

[jcv270079-bib-0013] Bringmann, L. F. , Albers, C. , Bockting, C. , Borsboom, D. , Ceulemans, E. , Cramer, A. , Epskamp, S. , Eronen, M. I. , Hamaker, E. , Kuppens, P. , Lutz, W. , McNally, R. J. , Molenaar, P. , Tio, P. , Voelkle, M. C. , & Wichers, M. (2022). Psychopathological networks: Theory, methods and practice. Behaviour Research and Therapy, 149, 104011. 10.1016/j.brat.2021.104011 34998034

[jcv270079-bib-0014] Burger, J. , Ralph‐Nearman, C. , & Levinson, C. A. (2022). Integrating clinician and patient case conceptualization with momentary assessment data to construct idiographic networks: Moving toward personalized treatment for eating disorders. Behaviour Research and Therapy, 159, 104221. 10.1016/j.brat.2022.104221 36327522

[jcv270079-bib-0015] Caspi, A. , Houts, R. M. , Belsky, D. W. , Goldman‐Mellor, S. J. , Harrington, H. , Israel, S. , Meier, M. H. , Ramrakha, S. , Shalev, I. , Poulton, R. , & Moffitt, T. E. (2014). The p factor: One general psychopathology factor in the structure of psychiatric disorders? Clinical Psychological Science, 2(2), 119–137. 10.1177/2167702613497473 25360393 PMC4209412

[jcv270079-bib-0016] Chaku, N. , & Beltz, A. M. (2022). Using temporal network methods to reveal the idiographic nature of development. Advances in Child Development and Behavior, 62, 159–190. 10.1016/bs.acdb.2021.11.003 35249681

[jcv270079-bib-0017] Conway, C. C. , Forbes, M. K. , & South, S. C. (2022). A hierarchical taxonomy of psychopathology (HiTOP) primer for mental health researchers. Clinical Psychological Science: A Journal of the Association for Psychological Science, 10(2), 236–258. 10.1177/21677026211017834 35599839 PMC9122089

[jcv270079-bib-0018] Cramer, A. O. J. , Waldorp, L. J. , Maas, H. L. J. V. D. , & Borsboom, D. (2010). Comorbidity: A network perspective. Behavioral and Brain Sciences, 33(2–3), 137–150. 10.1017/S0140525X09991567 20584369

[jcv270079-bib-0019] Crone, E. A. , & Dahl, R. E. (2012). Understanding adolescence as a period of social–affective engagement and goal flexibility. Nature Reviews Neuroscience, 13(9), 636–650. 10.1038/nrn3313 22903221

[jcv270079-bib-0020] Cusack, C. E. , Ralph‐Nearman, C. , Christian, C. , Fisher, A. J. , & Levinson, C. A. (2024). Understanding heterogeneity, comorbidity, and variability in depression: Idiographic models and depression outcomes. Journal of Affective Disorders, 356, 248–256. 10.1016/j.jad.2024.04.034 38608769

[jcv270079-bib-0021] Dalsgaard, S. , Thorsteinsson, E. , Trabjerg, B. B. , Schullehner, J. , Plana‐Ripoll, O. , Brikell, I. , Wimberley, T. , Thygesen, M. , Madsen, K. B. , Timmerman, A. , Schendel, D. , McGrath, J. J. , Mortensen, P. B. , & Pedersen, C. B. (2020). Incidence rates and cumulative incidences of the full spectrum of diagnosed mental disorders in childhood and adolescence. JAMA Psychiatry, 77(2), 155. 10.1001/jamapsychiatry.2019.3523 31746968 PMC6902162

[jcv270079-bib-0022] De Smet, M. M. , Schoofs, M. , Peeters, H. , Van Nieuwenhove, K. , & Meganck, R. (2024). Toward meaningful networks: How qualitative research can inform idiographic assessment and personalized care. Qualitative Psychology. 10.1037/qup0000314

[jcv270079-bib-0023] Do, Q. B. , McKone, K. M. P. , Hamilton, J. L. , Stone, L. B. , Ladouceur, C. D. , & Silk, J. S. (2023). The link between adolescent girls’ interpersonal emotion regulation with parents and peers and depressive symptoms: A real‐time investigation. Development and Psychopathology, 37, 1–15. 10.1017/S0954579423001359 37933501

[jcv270079-bib-0024] Du, X. , Skjerdingstad, N. , Freichel, R. , Ebrahimi, O. V. , Hoekstra, R. H. A. , & Epskamp, S. (2025). Moving from exploratory to confirmatory network analysis: An evaluation of structural equation modeling fit indices and cutoff values in network psychometrics. Psychological Methods. Advance online publication. 10.1037/met0000760 40549595

[jcv270079-bib-0025] Ebrahimi, O. V. (2023). Systems‐based thinking in psychology and the mental health sciences. Nature Reviews Psychology, 2(6), 6. 10.1038/s44159-023-00193-w PMC10171725 37304192

[jcv270079-bib-0026] Ebrahimi, O. V. , Borsboom, D. , Hoekstra, R. H. A. , Epskamp, S. , Ostinelli, E. G. , Bastiaansen, J. A. , & Cipriani, A. (2023). Towards precision in the diagnostic profiling of patients: Leveraging symptom dynamics in the assessment of major depressive disorder. PsyArXiv. 10.31234/osf.io/wh6cf PMC11039556 38584324

[jcv270079-bib-0027] Ebrahimi, O. V. , Burger, J. , Hoffart, A. , & Johnson, S. U. (2021). Within‐ and across‐day patterns of interplay between depressive symptoms and related psychopathological processes: A dynamic network approach during the COVID‐19 pandemic. BMC Medicine, 19(1), 317. 10.1186/s12916-021-02179-y 34844588 PMC8629696

[jcv270079-bib-0028] Eisenberg, N. , Cumberland, A. , Spinrad, T. L. , Fabes, R. A. , Shepard, S. A. , Reiser, M. , Murphy, B. C. , Losoya, S. H. , & Guthrie, I. K. (2001). The relations of regulation and emotionality to children’s externalizing and internalizing problem behavior. Child Development, 72(4), 1112–1134. 10.1111/1467-8624.00337 11480937

[jcv270079-bib-0029] Enders, C. K. , & Bandalos, D. L. (2001). The relative performance of full information maximum likelihood estimation for missing data in structural equation models. Structural Equation Modeling: A Multidisciplinary Journal, 8(3), 430–457. 10.1207/S15328007SEM0803_5

[jcv270079-bib-0030] Epskamp, S. (2020). Psychometric network models from time‐series and panel data. Psychometrika, 85(1), 206–231. 10.1007/s11336-020-09697-3 32162233 PMC7186258

[jcv270079-bib-0031] Epskamp, S. , Borsboom, D. , & Fried, E. I. (2018). Estimating psychological networks and their accuracy: A tutorial paper. Behavior Research Methods, 50(1), 195–212. 10.3758/s13428-017-0862-1 28342071 PMC5809547

[jcv270079-bib-0032] Epskamp, S. , Cramer, A. O. J. , Waldorp, L. J. , Schmittmann, V. D. , & Borsboom, D. (2012). qgraph: Network visualizations of relationships in psychometric data. Journal of Statistical Software, 48(4), 1–18. 10.18637/jss.v048.i04

[jcv270079-bib-0033] Epskamp, S. , & Isvoranu, A.‐M. (2022). New trends in network modeling of psychopathology. World Psychiatry, 21(3), 463–464. 10.1002/wps.21017 36073689 PMC9453883

[jcv270079-bib-0034] Flouri, E. , Papachristou, E. , Midouhas, E. , Ploubidis, G. B. , Lewis, G. , & Joshi, H. (2019). Developmental cascades of internalising symptoms, externalising problems and cognitive ability from early childhood to middle adolescence. European Psychiatry, 57, 61–69. 10.1016/j.eurpsy.2018.12.005 30677550

[jcv270079-bib-0035] Fraser, A. , Macdonald‐Wallis, C. , Tilling, K. , Boyd, A. , Golding, J. , Davey Smith, G. , Henderson, J. , Macleod, J. , Molloy, L. , Ness, A. , Ring, S. , Nelson, S. M. , & Lawlor, D. A. (2013). Cohort profile: The Avon Longitudinal Study of Parents and Children: ALSPAC mothers cohort. International Journal of Epidemiology, 42(1), 97–110. 10.1093/ije/dys066 22507742 PMC3600619

[jcv270079-bib-0036] Freichel, R. (2023). Symptom network analysis tools for applied researchers with cross‐sectional and panel data – A brief overview and multiverse analysis. Psychological Reports, 128(6), 00332941231213649. 10.1177/00332941231213649 PMC12480605 37944560

[jcv270079-bib-0037] Freichel, R. , & Epskamp, S. (2024). Handling problematic between‐person estimates in panel network models: A comparative simulation study. OSF. 10.31234/osf.io/eqaux

[jcv270079-bib-0038] Freichel, R. , Pfirrmann, J. , De Jong, P. J. , Cousijn, J. , Franken, I. H. A. , Oldehinkel, A. J. , Veer, I. M. , & Wiers, R. W. (2024). Executive functioning, internalizing and externalizing symptoms: Understanding developmental dynamics through panel network approaches. JAACAP Open, 2(1), 66–77. 10.1016/j.jaacop.2023.11.001 39554700 PMC11562421

[jcv270079-bib-0064] Freichel, R. , Skjerdingstad, N. , Mansueto, A. C. , Epskamp, S. , Hoffart, A. , Johnson, S. U. , & Ebrahimi, O. V. (2025). Use of substances to cope predicts posttraumatic stress disorder symptom persistence: Investigating patterns of interactions between symptoms and its maintaining mechanisms. Psychological Trauma: Theory, Research, Practice, and Policy, 17(1), 216–224. 10.1037/tra0001624 38059942

[jcv270079-bib-0039] Fried, E. I. (2022). Studying mental health problems as systems, not syndromes. Current Directions in Psychological Science, 31(6), 096372142211140. 10.1177/09637214221114089

[jcv270079-bib-0040] Fried, E. I. , & Cramer, A. O. J. (2017). Moving forward: Challenges and directions for psychopathological network theory and methodology. Perspectives on Psychological Science: A Journal of the Association for Psychological Science, 12(6), 999–1020. 10.1177/1745691617705892 28873325

[jcv270079-bib-0041] Fried, E. I. , & Robinaugh, D. J. (2020). Systems all the way down: Embracing complexity in mental health research. BMC Medicine, 18(1), 205. 10.1186/s12916-020-01668-w 32660482 PMC7359484

[jcv270079-bib-0042] Goodman, A. , & Goodman, R. (2009). Strengths and difficulties questionnaire as a dimensional measure of child mental health. Journal of the American Academy of Child & Adolescent Psychiatry, 48(4), 400–403. 10.1097/CHI.0b013e3181985068 19242383

[jcv270079-bib-0043] Haslbeck, J. M. B. , & Ryan, O. (2022). Recovering within‐person dynamics from psychological time series. Multivariate Behavioral Research, 57(5), 735–766. 10.1080/00273171.2021.1896353 34154483

[jcv270079-bib-0044] Hoekstra, R. H. A. , Epskamp, S. , Nierenberg, A. A. , Borsboom, D. , & McNally, R. J. (2024). Testing similarity in longitudinal networks: The Individual Network Invariance Test. Psychological Methods. 10.1037/met0000638 38602781

[jcv270079-bib-0045] Höltge, J. , Theron, L. , & Ungar, M. (2022). A multisystemic perspective on the temporal interplay between adolescent depression and resilience‐supporting individual and social resources. Journal of Affective Disorders, 297, 225–232. 10.1016/j.jad.2021.10.030 34695498

[jcv270079-bib-0046] Johal, S. K. , & Rhemtulla, M. (2023). Comparing estimation methods for psychometric networks with ordinal data. Psychological Methods, 28(6), 1251–1272. 10.1037/met0000449 34928677

[jcv270079-bib-0047] Johnson, D. , Dupuis, G. , Piche, J. , Clayborne, Z. , & Colman, I. (2018). Adult mental health outcomes of adolescent depression: A systematic review. Depression and Anxiety, 35(8), 700–716. 10.1002/da.22777 29878410

[jcv270079-bib-0048] Kieling, C. , Buchweitz, C. , Caye, A. , Silvani, J. , Ameis, S. H. , Brunoni, A. R. , Cost, K. T. , Courtney, D. B. , Georgiades, K. , Merikangas, K. R. , Henderson, J. L. , Polanczyk, G. V. , Rohde, L. A. , Salum, G. A. , & Szatmari, P. (2024). Worldwide prevalence and disability from mental disorders across childhood and adolescence: Evidence from the global burden of disease study. JAMA Psychiatry, 81(4), 347. 10.1001/jamapsychiatry.2023.5051 38294785 PMC10831630

[jcv270079-bib-0049] Liu, K. , Thompson, R. C. , Watson, J. , Montena, A. L. , & Warren, S. L. (2023). Developmental trajectories of internalizing and externalizing symptoms in youth and associated gender differences: A directed network perspective. Research on Child and Adolescent Psychopathology, 51(11), 1627–1639. 10.1007/s10802-023-01106-4 37548898 PMC10627904

[jcv270079-bib-0050] Masten, A. S. , & Cicchetti, D. (2010). Developmental cascades. Development and Psychopathology, 22(3), 491–495. 10.1017/S0954579410000222 20576173

[jcv270079-bib-0051] McLaughlin, K. A. , Garrad, M. C. , & Somerville, L. H. (2015). What develops during emotional development? A component process approach to identifying sources of psychopathology risk in adolescence. Dialogues in Clinical Neuroscience, 17(4), 403–410. 10.31887/DCNS.2015.17.4/kmclaughlin 26869841 PMC4734878

[jcv270079-bib-0052] Mircioiu, C. , & Atkinson, J. (2017). A comparison of parametric and non‐parametric methods applied to a Likert scale. Pharmacy: Journal of Pharmacy, Education and Practice, 5(2), 26. 10.3390/pharmacy5020026 28970438 PMC5597151

[jcv270079-bib-0053] Moilanen, K. L. , Shaw, D. S. , & Maxwell, K. L. (2010). Developmental cascades: Externalizing, internalizing, and academic competence from middle childhood to early adolescence. Development and Psychopathology, 22(3), 635–653. 10.1017/S0954579410000337 20576184 PMC3168570

[jcv270079-bib-0054] Olivier, E. , Morin, A. J. S. , Tardif‐Grenier, K. , Archambault, I. , Dupéré, V. , & Hébert, C. (2022). Profiles of anxious and depressive symptoms among adolescent boys and girls: Associations with coping strategies. Journal of Youth and Adolescence, 51(3), 570–584. 10.1007/s10964-022-01572-x 35038084

[jcv270079-bib-0055] Pasman, J. A. , Meijsen, J. J. , Haram, M. , Kowalec, K. , Harder, A. , Xiong, Y. , Nguyen, T.‐D. , Jangmo, A. , Shorter, J. R. , Bergstedt, J. , Das, U. , Zetterberg, R. , Tate, A. , Lichtenstein, P. , Larsson, H. , Odsbu, I. , Werge, T. , Reichborn‐Kjennerud, T. , Andreassen, O. A. , … Lu, Y. (2023). Epidemiological overview of major depressive disorder in Scandinavia using nationwide registers. The Lancet Regional Health ‐ Europe, 29, 100621. 10.1016/j.lanepe.2023.100621 37265784 PMC10230616

[jcv270079-bib-0063] R Core Team . (2024). R: A language and environment for statistical computing. R Foundation for Statistical Computing. https://www.R‐project.org/

[jcv270079-bib-0056] Robitzsch, A. (2020). Why ordinal variables can (almost) always be treated as continuous variables: Clarifying assumptions of robust continuous and ordinal factor analysis estimation methods. Frontiers in Education, 5, 589965. 10.3389/feduc.2020.589965

[jcv270079-bib-0057] Russell, M. A. , & Gajos, J. M. (2020). Annual research review: Ecological momentary assessment studies in child psychology and psychiatry. The Journal of Child Psychology and Psychiatry and Allied Disciplines, 61(3), 376–394. 10.1111/jcpp.13204 31997358 PMC8428969

[jcv270079-bib-0058] Speyer, L. G. , Eisner, M. , Ribeaud, D. , Luciano, M. , Auyeung, B. , & Murray, A. L. (2021). Developmental relations between internalising problems and ADHD in childhood: A symptom level perspective. Research on Child and Adolescent Psychopathology, 49(12), 1567–1579. 10.1007/s10802-021-00856-3 34363556 PMC8557182

[jcv270079-bib-0059] Speyer, L. G. , Hall, H. A. , Ushakova, A. , Luciano, M. , Auyeung, B. , & Murray, A. L. (2022). Within‐person relations between domains of socio‐emotional development during childhood and adolescence. Research on Child and Adolescent Psychopathology, 50(10), 1261–1274. 10.1007/s10802-022-00933-1 35670883 PMC9606067

[jcv270079-bib-0060] Speyer, L. G. , Ushakova, A. , Hall, H. A. , Luciano, M. , Auyeung, B. , & Murray, A. L. (2022). Analyzing dynamic change in children’s socioemotional development using the strengths and difficulties questionnaire in a large United Kingdom longitudinal study. Journal of Psychopathology and Clinical Science, 131(2), 162–171. 10.1037/abn0000714 34928626

[jcv270079-bib-0061] Thapar, A. , Eyre, O. , Patel, V. , & Brent, D. (2022). Depression in young people. The Lancet, 400(10352), 617–631. 10.1016/S0140-6736(22)01012-1 35940184

[jcv270079-bib-0062] UNICEF (Red.) . (2021). On my mind: Promoting, protecting and caring for children’s mental health. UNICEF.

