# Supplementary material for: Developmental dynamics of symptoms of emotional problems in childhood and adolescence: A longitudinal network analysis
Source: JCPP Adv. 2025 Nov 28;6(3):e70079. doi: 10.1002/jcv2.70079 (PMC13339606; doi:10.1002/jcv2.70079)
Supplement: Supplementary file 1 [file JCV2-6-e70079-s001.docx]

**Developmental Dynamics of Symptoms of Emotional Problems in Childhood and Adolescence: A Longitudinal Network Analysis**

**Supporting Information**

Table of Content

[Table S1. Age Summary Statistics across timepoints	2](#_Toc213676973)

[Table S2a. Missingness Analysis Table	2](#_Toc213676974)

[Table S2b. Missingness Analysis Table	3](#_Toc213676975)

[Table S3. Descriptive statistics timepoint 1	3](#_Toc213676976)

[Table S4. Descriptive statistics timepoint 2	4](#_Toc213676977)

[Table S5. Descriptive statistics timepoint 3	4](#_Toc213676978)

[Table S6. Descriptive statistics timepoint 4	5](#_Toc213676979)

[Table S7. Descriptive statistics timepoint 5	5](#_Toc213676980)

[Table S8. Descriptive statistics timepoint 6	6](#_Toc213676981)

[Table S9. Descriptive statistics timepoint 7	7](#_Toc213676982)

[Table S10. ICD-10 Diagnostic Summary by sex	7](#_Toc213676983)

[Table S11. Percentage of children in each SDQ Emotional Symptoms category by sex and timepoint	8](#_Toc213676984)

[Table S12. Cronbach’s Alpha Estimates Across Timepoints	9](#_Toc213676985)

[Figure S1. Distribution of participants by number of complete SDQ timepoints.	10](#_Toc213676986)

Appendix S1. [Fit-indices	11](#_Toc213676987)

[Figure S2. Strength Centrality of Temporal Pruned Model	12](#_Toc213676988)

[Figure S3. Strength Centrality of Contemporaneous Pruned Model	13](#_Toc213676989)

[Figure S4. Bootstrapping Results: Percentage of Edge Presence in Temporal Network	13](#_Toc213676990)

[Figure S5. Bootstrapping Results: Percentage of Edge Presence in Contemporaneous Network	14](#_Toc213676991)

[Figure S6. Distribution of item 1 across timepoints for males and females	15](#_Toc213676992)

[Figure S7. Distribution of item 2 across timepoints for males and females	15](#_Toc213676993)

[Figure S8. Distribution of item 3 across timepoints for males and females	16](#_Toc213676994)

[Figure S9. Distribution of item 4 across timepoints for males and females	16](#_Toc213676995)

[Figure S10. Distribution of item 5 across timepoints for males and females	17](#_Toc213676996)

[References	17](#_Toc213676997)

### Table S1. Age Summary Statistics across timepoints

| Timepoint | Min_Age | Max_Age | Mean_Age | Mean_Age_Diff |
| --- | --- | --- | --- | --- |
| age_tp1 | 3.67 | 5.35 | 4.00 |  |
| age_tp2 | 6.67 | 8.42 | 6.79 | 2.79 |
| age_tp3 | 7.83 | 14.00 | 8.20 | 1.41 |
| age_tp4 | 9.50 | 11.00 | 9.65 | 1.45 |
| age_tp5 | 11.42 | 13.83 | 11.72 | 2.07 |
| age_tp6 | 12.83 | 16.08 | 13.16 | 1.44 |
| age_tp7 | 16.50 | 18.33 | 16.84 | 3.68 |

### Table S2a. Missingness Analysis Table

| TP1.Observations | TP1 %Missing | TP2.Observations | TP2 % Missing | TP3.Observations | TP3%. Missing | TP4.Observations | TP4. %Missing |
| --- | --- | --- | --- | --- | --- | --- | --- |
| 9,338 | 39.53 | 8,280 | 46.38 | 7,688 | 50.22 | 7,902 | 48.83 |
| 9,339 | 39.53 | 8,253 | 46.56 | 7,676 | 50.29 | 7,828 | 49.31 |
| 9,362 | 39.38 | 8,295 | 46.29 | 7,666 | 50.36 | 7,898 | 48.86 |
| 9,349 | 39.46 | 8,275 | 46.42 | 7,674 | 50.31 | 7,932 | 48.64 |
| 9,345 | 39.49 | 8,254 | 46.55 | 7,696 | 50.17 | 7,872 | 49.03 |

### Table S2b. Missingness Analysis Table

| TP5.Observations | TP5%Missing | TP6.Observations | TP6%. Missing | TP7.Observations | TP7. %Missing |
| --- | --- | --- | --- | --- | --- |
| 7,205 | 53.34 | 6,956 | 54.96 | 5,575 | 63.90 |
| 7,156 | 53.66 | 6,908 | 55.27 | 5,501 | 64.38 |
| 7,206 | 53.34 | 6,930 | 55.13 | 5,554 | 64.04 |
| 7,200 | 53.38 | 6,940 | 55.06 | 5,550 | 64.06 |
| 7,179 | 53.51 | 6,908 | 55.27 | 5,495 | 64.42 |

### Table S3. Descriptive statistics timepoint 1

| Variable | Mean  Boys Girls | | SD  Boys Girls | | Median | | NA  Boys Girls | |
| --- | --- | --- | --- | --- | --- | --- | --- | --- |
| Item 1 | 1.187 | 1.243 | 0.44 | 0.49 | 1.00 | 1.00 | 2795 | 2788 |
| Item 2 | 1.155 | 1.132 | 0.41 | 0.38 |  |  | 2796 | 2786 |
| Item 3 | 1.151 | 1.157 | 0.39 | 0.39 |  |  | 2788 | 2771 |
| Item 4 | 1.604 | 1.604 | 0.68 | 0.67 |  |  | 2798 | 2774 |
| Item 5 | 1.329 | 1.342 | 0.54 | 0.54 |  |  | 2799 | 2777 |

### Table S4. Descriptive statistics timepoint 2

| Variable | Mean  Boys Girls | | SD  Boys Girls | | Median | | NA  Boys Girls | |
| --- | --- | --- | --- | --- | --- | --- | --- | --- |
| Item 1 | 1.293 | 1.386 | 0.54 | 0.59 | 1.00 | 1.00 | 3384 | 3274 |
| Item 2 | 1.295 | 1.29 | 0.53 | 0.51 |  |  | 3402 | 3285 |
| Item 3 | 1.165 | 1.177 | 0.41 | 0.41 |  |  | 3374 | 3269 |
| Item 4 | 1.415 | 1.455 | 0.61 | 0.61 |  |  | 3379 | 3284 |
| Item 5 | 1.267 | 1.273 | 0.51 | 0.5 |  |  | 3388 | 3296 |

### Table S5. Descriptive statistics timepoint 3

| Variable | Mean  Boys Girls | | SD  Boys Girls | | Median | | NA  Boys Girls | |
| --- | --- | --- | --- | --- | --- | --- | --- | --- |
| Item 1 | 1.309 | 1.407 | 0.54 | 0.6 | 1.00 | 1.00 | 3740 | 3517 |
| Item 2 | 1.365 | 1.345 | 0.56 | 0.54 |  |  | 3744 | 3525 |
| Item 3 | 1.205 | 1.231 | 0.46 | 0.47 |  |  | 3751 | 3528 |
| Item 4 | 1.423 | 1.456 | 0.62 | 0.61 |  |  | 3746 | 3525 |
| Item 5 | 1.31 | 1.319 | 0.54 | 0.53 |  |  | 3735 | 3514 |

### Table S6. Descriptive statistics timepoint 4

| Variable | Mean  Boys Girls | | SD  Boys Girls | | Median | | NA  Boys Girls | |
| --- | --- | --- | --- | --- | --- | --- | --- | --- |
| Item 1 | 1.358 | 1.501 | 0.58 | 0.65 | 1.00 | 1.00 | 3655 | 3393 |
| Item 2 | 1.292 | 1.304 | 0.52 | 0.52 |  |  | 3686 | 3437 |
| Item 3 | 1.164 | 1.197 | 0.4 | 0.44 |  |  | 3658 | 3394 |
| Item 4 | 1.331 | 1.392 | 0.56 | 0.58 |  |  | 3636 | 3380 |
| Item 5 | 1.236 | 1.259 | 0.49 | 0.51 |  |  | 3655 | 3421 |

### Table S7. Descriptive statistics timepoint 5

| Variable | Mean  Boys Girls | | SD  Boys Girls | | Median | | NA  Boys Girls | |
| --- | --- | --- | --- | --- | --- | --- | --- | --- |
| Item 1 | 1.343 | 1.502 |  | 0.64 | 1.00 | 1.00 | 4047 | 3704 |
| Item 2 | 1.314 | 1.342 |  | 0.55 |  |  | 4078 | 3721 |
| Item 3 | 1.152 | 1.201 |  | 0.45 |  |  | 4046 | 3703 |
| Item 4 | 1.3 | 1.329 |  | 0.55 |  |  | 4051 | 3704 |
| Item 5 | 1.217 | 1.231 |  | 0.48 |  |  | 4060 | 3716 |

### Table S8. Descriptive statistics timepoint 6

| Variable | Mean  Boys Girls | | SD  Boys Girls | | Median | | NA  Boys Girls | |
| --- | --- | --- | --- | --- | --- | --- | --- | --- |
| Item 1 | 1.371 | 1.561 | 0.58 | 0.65 | 1.00 | 1.00 | 4183 | 3817 |
| Item 2 | 1.262 | 1.311 | 0.5 | 0.53 |  |  | 4207 | 3841 |
| Item 3 | 1.148 | 1.201 | 0.4 | 0.46 |  |  | 4194 | 3832 |
| Item 4 | 1.287 | 1.341 | 0.53 | 0.56 |  |  | 4193 | 3823 |
| Item 5 | 1.162 | 1.207 | 0.42 | 0.46 |  |  | 4206 | 3842 |

### Table S9. Descriptive statistics timepoint 7

| Variable | Mean  Boys Girls | | SD  Boys Girls | | Median | | NA  Boys Girls | |
| --- | --- | --- | --- | --- | --- | --- | --- | --- |
| Item 1 | 1.3 | 1.608 | 0.55 | 0.7 | 1.00 | 1.00 | 4959 | 4439 |
| Item 2 | 1.323 | 1.465 | 0.56 | 0.63 |  |  | 5011 | 4462 |
| Item 3 | 1.114 | 1.262 | 0.37 | 0.52 |  |  | 4977 | 4442 |
| Item 4 | 1.237 | 1.319 | 0.51 | 0.56 |  |  | 4969 | 4454 |
| Item 5 | 1.106 | 1.208 | 0.35 | 0.47 |  |  | 5006 | 4473 |

### Table S10. ICD-10 Diagnostic Summary by sex

| Variable | N (Male) | % Diagnosis (Male) | N (Female) | % Diagnosis (Female) |
| --- | --- | --- | --- | --- |
| Anxiety Disorder (7y) | 4,143 | 2.0 | 3,915 | 1.4 |
| Depression (7y) | 4,058 | 0.7 | 3,850 | 0.5 |
| Any Emotional Disorder (7y) | 4,143 | 2.5 | 3,915 | 1.7 |
| Anxiety Disorder (10y) | 3,857 | 2.1 | 3,798 | 2.1 |
| Depression (10y) | 3,772 | 1.0 | 3,725 | 0.9 |
| Any Emotional Disorder (10y) | 3,857 | 2.8 | 3,798 | 2.8 |
| Anxiety Disorder (13y) | 3,488 | 1.4 | 3,467 | 1.6 |
| Depression (13y) | 3,401 | 0.9 | 3,411 | 0.7 |
| Any Emotional Disorder (13y) | 3,488 | 1.9 | 3,467 | 2.0 |

*Note***.** The table presents the number of participants (N) and percentage (%) with an ICD-10 diagnosis of any emotional disorder, any anxiety disorder or depressive disorder at ages 7, 10 and 13, stratified by sex. Percentages represent the proportion of individuals with a diagnosis (coded as 1) among those with available data at each timepoint. Diagnoses were based on the Development and Well-Being Assessment (DAWBA) and coded according to ICD-10 criteria.

### Table S11. Percentage of children in each SDQ Emotional Symptoms category by sex and timepoint

| TP | Male | | | Female | | |
| --- | --- | --- | --- | --- | --- | --- |
|  | Normal | Borderline | Abnormal | Normal | Borderline | Abnormal |
| TP 1 | 89.5 | 5.9 | 4.6 | 89.6 | 5.9 | 4.5 |
| TP 2 | 84.7 | 6.6 | 8.7 | 83.0 | 7.6 | 9.4 |
| TP 3 | 88.2 | 5.6 | 6.2 | 86.1 | 7.0 | 6.9 |
| TP 4 | 88.5 | 5.3 | 6.3 | 84.4 | 7.4 | 8.2 |
| TP 5 | 88.9 | 4.9 | 6.2 | 86.1 | 6.0 | 8.0 |
| TP 6 | 90.0 | 4.8 | 5.2 | 85.3 | 6.7 | 8.0 |
| TP 7 | 91.7 | 3.7 | 4.7 | 81.3 | 7.3 | 11.4 |

*Note*: Values represent the percentage of male and female participants classified as Normal, Borderline, or Abnormal based on the Strengths and Difficulties Questionnaire (SDQ) Emotional Symptoms subscale at each timepoint.

### Table S12. Cronbach’s Alpha Estimates Across Timepoints

|  | Cronbach’s Alpha |
| --- | --- |
| Timepoint 1 | 0.55 |
| Timepoint 2 | 0.63 |
| Timepoint 3 | 0.69 |
| Timepoint 4 | 0.68 |
| Timepoint 5 | 0.67 |
| Timepoint 6 | 0.67 |
| Timepoint 7 | 0.71 |

### Figure S1. Distribution of participants by number of complete SDQ timepoints.

*Note*. Participants with zero complete timepoints were excluded.

### Appendix S1. Fit-indices

Previous work (Speyer et al., 2022) has discussed whether the CFI and TLI are the most suited indices to apply when analysing data like the SDQ, where the average correlation between items is low. This is due to only small differences between the theoretical and the null model, entailing low CFI and TLI values, but not affecting the RMSEA (Gomez & Stavropoulos, 2019).

### Figure S2. Strength Centrality of Temporal Pruned Model

### Figure S3. Strength Centrality of Contemporaneous Pruned Model

### Figure S4. Bootstrapping Results: Percentage of Edge Presence in Temporal Network

*Note.* The numbers displayed in the figure represent the percentage of edge presence during the computation of 1000 bootstraps (with a random 75% of the sample in each iteration). The lower triangular of the figure refers to directed associations outgoing from the variable in the respective row towards the variable in the respective column.

### Figure S5. Bootstrapping Results: Percentage of Edge Presence in Contemporaneous Network

*Note.* The figure refers to the undirected contemporaneous associations between different variables.

### Figure S6. Distribution of item 1 across timepoints for males and females

### Figure S7. Distribution of item 2 across timepoints for males and females

### Figure S8. Distribution of item 3 across timepoints for males and females

### Figure S9. Distribution of item 4 across timepoints for males and females

### Figure S10. Distribution of item 5 across timepoints for males and females

### References

Gomez, R., & Stavropoulos, V. (2019). Parent Ratings of the Strengths and Difficulties Questionnaire: What Is the Optimum Factor Model? *Assessment*, *26*(6), 1142–1153. https://doi.org/10.1177/1073191117721743

Speyer, L. G., Ushakova, A., Hall, H. A., Luciano, M., Auyeung, B., & Murray, A. L. (2022). Analyzing dynamic change in children’s socioemotional development using the strengths and difficulties questionnaire in a large United Kingdom longitudinal study. *Journal of Psychopathology and Clinical Science*, *131*(2), 162–171. https://doi.org/10.1037/abn0000714
